# Short-term dietary deoxynivalenol exposure negatively affects performance, intestinal and reproductive functions in laying hens

**DOI:** 10.1038/s41598-026-46100-0

**Published:** 2026-04-11

**Authors:** E. Basili, L. Star, G. Beleva, F. Molist, A. Milanova, R. R. Santos

**Affiliations:** 1 Department of Research and Development, Schothorst Feed Research, PO Box 533, 8200 AM Lelystad, The Netherlands; 2 Department of Pharmacology, Animal Physiology, Biochemistry and Chemistry, Faculty of Veterinary Medicine, Trakia University, 6000 Stara Zagora, Bulgaria

**Keywords:** Mycotoxins, Intestine, Shell gland, Ovary, Poultry, Environment, Biochemistry, Diseases, Environmental sciences, Physiology, Zoology

## Abstract

The detrimental effects of deoxynivalenol (DON) on broiler chickens are well documented; however, studies on laying hens fed diets naturally contaminated with this mycotoxin remain limited. To evaluate the impact of short-term DON exposure, 600 56-week-old Dekalb White laying hens were fed either a marginally contaminated control diet (CON) or a diet containing 2.45 mg/kg DON for 16 weeks. Production performance was impaired by DON as early as 8 weeks of exposure. Although no changes were observed in the physical characteristics of the eggshells, mRNA expression analyses revealed disruptions in calcium and phosphorus metabolism. Despite similar egg production between treatments, DON exposure led to degeneration of primordial, primary, and large white follicles. Metabolomic analysis of the excreta indicated altered lipid metabolism, suggestive of intestinal inflammation, along with increased hydrogen sulphide (H_2_S) levels. In conclusion, short-term exposure to DON negatively affects intestinal function, reproductive health, and feed efficiency in laying hens.

**Supplementary Information:**

The online version contains supplementary material available at 10.1038/s41598-026-46100-0.

## Introduction

A longitudinal study based on experiments conducted over the past decades has demonstrated that broiler chickens are sensitive to the *Fusarium* mycotoxin deoxynivalenol (DON)^1^. The European Food Safety Authority (EFSA) suggested to reduce the reference point of DON in broiler diets, reduced from 5 to 0.6 mg/kg^2^. However, because of the limited data available for laying hens, particularly at low and realistic exposure levels, the maximum recommended level of DON for this category remains 5 mg/kg. In a study comparing the sensitivity to DON exposure of the white strain Lohmann Selected Leghorn and the brown strain Lohmann Brown, starting at 23 weeks of age, only the Lohmann Brown hens exposed to 9.9 mg/kg DON showed impaired eggshell quality at 40 and 60 weeks^3^. Moreover, these authors reported a significant decrease in the laying rate of Lohmann Brown hens after 12 weeks of exposure. A short-term (6-week) exposure of 16-week-old Jing Tint laying hens to 9 mg/kg DON resulted in reduced weight gain, decreased jejunal villus height (VH), and increased crypt depth (CD)^4^. When the same authors exposed the hens to 4.5 mg/kg DON, the VH: CD ratio decreased, and the relative mRNA expression of the tight junction proteins claudin-1 (CLDN1) and claudin-5 (CLDN5), as well as the host defence peptides avian beta-defensin 10, avian beta-defensin 11, and cathelicidin 2, was down-regulated. Exposure to DON affects not only mucus production by intestinal cells—evidenced by the down-regulation of mucin-2 (MUC2)^5^—but also cellular lipid metabolism through the down-regulation of fatty acid binding proteins (FABP) and acetyl-CoA carboxylase (ACC). Damage to the duodenum caused by DON exposure is expected to reduce the uptake of calcium (Ca) and phosphorus (P) because this segment of the small intestine is the primary site of their absorption^6^, which may lead to impaired egg production and quality. It is well established that, particularly in aged layers, reduced Ca absorption results in poorer eggshell quality^7^.

The Ca absorbed in the duodenal epithelium binds to calbindin-D28k (CaBP28k) and, with the help of plasma membrane Ca^2+^-ATPase 1b (PMCA1b), enters the bloodstream^6^. P absorption is regulated by the sodium–phosphate co-transporter 2b (NPt2b), which adjusts according to dietary P levels^8^. The mobilisation of Ca is controlled by the extracellular Ca-sensing receptor (CaSR) and the vitamin D receptor (VDR)^9^, while the xenotropic and polytropic retrovirus receptor 1 (XPR1) facilitates the efflux of P from cells^10^. During eggshell formation, osteopontin (OPN) is synthesised by various cell types, including those in the eggshell gland during calcification^11^. Ovocleidin-17 (OC17) initiates eggshell calcification, ovocalyxin-32 (OCX32) contributes to the formation of the outer layers, and ovocalyxin-36 (OCX36) is involved in forming the inner layers^12^. Therefore, if DON causes damage to the duodenum, it is expected to disrupt this network of processes associated with eggshell formation and quality.

The negative impact of DON on reproduction has been demonstrated both in vitro^13^ and in vivo^14^ in mammalian species. Furthermore, feeding 20- to 23-week-old White Leghorn hens diets containing oats naturally contaminated with DON (2.5, 3.1, and 4.9 mg/kg in the final diet) for 10 weeks, followed by artificial insemination, resulted in a higher incidence of malformed embryos and delayed foetal development—characterised by unabsorbed yolk sacs and delayed ossification—regardless of the dietary DON concentration^15^. Endoplasmic reticulum stress induced by DON has been linked to apoptosis in porcine ovarian cells, inhibiting the release of insulin-like growth factor 1 (IGF1)^16^. In laying hens, IGF1, together with growth hormone (GH), plays a key role in egg production^17^. Apoptosis may also reduce the ovarian follicular population through the up-regulation of pro-apoptotic proteins such as caspase (CASP) and the down-regulation of anti-apoptotic proteins such as beclin-2 (BCL2)^18^. Moreover, the increased occurrence of mycotoxin contamination in crops due to climate change^19^ and the consequent rise in mycotoxin exposure may impair nutrient absorption, potentially leading to higher gas emissions through increased excretion of nitrogen (N) and hydrogen sulphide (H_2_S)—by-products of microbial decomposition of caecal fermented nitrogen compounds and undigested sulphur proteins, respectively^20^.

In the present study, we evaluated the effects of dietary exposure to DON over 8 and 16 weeks on production performance and on the morphology and function of the duodenum, shell gland, and ovary in 56-week-old laying hens. Dekalb White hens were fed diets that were either marginally or naturally *Fusarium*-contaminated, with DON as the predominant mycotoxin. Production performance and egg quality were monitored throughout the feeding period. In addition, intestinal, shell gland, and ovarian functions were analysed after 8 and 16 weeks of exposure—corresponding to 64 and 72 weeks of age, respectively. The duodenum and shell gland were subjected to histological analysis, including Ca detection in intestinal and shell gland cells, along with quantitative real-time polymerase chain reaction (qRT-PCR) analyses. Ovarian hierarchical follicles were counted, and the proportions of normal preantral and prehierarchical follicles were determined. Serum concentrations of DON, Ca, P, glucose, and liver function markers were measured. Finally, a non-invasive metabolomic analysis of droppings was conducted to identify indicators of intestinal function.

## Results

### Analysis of the diets

The DON diet was prepared using naturally contaminated maize, resulting in a multi-contaminated feed in which DON was the predominant mycotoxin (Table 1). The levels of lutein, xanthophylls, and zeaxanthin were higher in the control (CON) diet than in the DON diet, while β-carotene levels were below the limit of quantification in both (Table 2).

**Table 1:** Levels of mycotoxins in each diet.

Mycotoxin	CON	DON
	Mycotoxin level (mg/kg)	Mycotoxin level (mg/kg)
DON	–	2.45
3 + 15 ADON	–	0.07
DON-3G	0.08	0.84
ZEN	–	0.21
FB_1_ + FB_2_	–	0.02
BEA	0.01	0.01

Analysed mycotoxins with their respective limits of quantification: Aflatoxin B_1_ (1 µg/kg), Aflatoxin B_2_ (1 µg/kg), Aflatoxin G_1_ (1 µg/kg), Aflatoxin G_2_ (1 µg/kg), Alternariol (2 µg/kg), Alternariol monomethyl ether (2 µg/kg), Beauvericin (BEA; 5 µg/kg), Citrinin (10 µg/kg), Cytochalasine E (2 µg/kg), Deoxynivalenol (DON; 20 µg/kg), 3 + 15 Ac-deoxynivalenol (3 + 15 ADON; 20 µg/kg), Deoxynivalenol-3-glucoside (DON-3G; 20 µg/kg), Diacetoxyscirpenol (5 µg/kg), Enniatin A (5 µg/kg), Enniatin A1 (5 µg/kg), Enniatin B (5 µg/kg), Enniatin B1 (5 µg/kg), Fumonisins B_1_ + B_2_ (FB_1_ + FB_2_; 20 µg/kg), Moniliformin (5 µg/kg), Nivalenol (50 µg/kg), Ochratoxin A (1 µg/kg), Roquefortine C (5 µg/kg), Sterigmatocystin (1 µg/kg), T-2/HT-2 toxin (10 µg/kg), Zearalenone (ZEN; 15 µg/kg).

CON: control diet; DON diet: diet contaminated with 2.45 mg/kg DON.

**Table 2:** Levels of carotenoids in each diet.

Carotenoid	CON	DON
	Carotenoid level (mg/kg)	Carotenoid level (mg/kg)
β-carotene	> 0.1	> 0.1
Lutein	4.9	3.1
Xanthophylls	10.9	7.1
Zeaxanthin	3.2	1.9

Limit of quantification: 1 mg/kg.

CON: Control diet; DON diet: diet contaminated with 2.45 mg/kg DON.

### Production performance

The experimental diets had no effect on the body weight (BW) of the laying hens. From 64 to 68 weeks of age, feed intake (FI) was significantly higher in hens fed the DON diet than in hens in the CON group. This increase in FI did not lead to higher laying rate, egg weight, or egg mass, but it did result in a significant rise in the feed conversion ratio (FCR), which was already elevated during weeks 60–64. Although no significant differences in performance were observed during the final 4 weeks of the trial (68–72 weeks of age), the overall FCR across the full experimental period (56–72 weeks of age) was significantly impaired (Table 3).

**Table 3:** Productive performance of laying hens fed control or DON-contaminated diets.

	CON	DON	SEM	*p*-value
BW (g)				
Week 56	1695	1688	13.5	0.74
Week 64	1715	1750	12.0	0.07
Week 72	1744	1749	12.9	0.78
FI (g/h/d)				
Week 56–60	130.4	129.8	1.65	0.80
Week 60–64	132.9	136.5	1.60	0.15
Week 64–68	138.7	144.5	1.54	0.03
Week 68–72	143.7	147.4	1.55	0.14
Week 56–72	136.4	139.1	1.09	0.11
FCR (FI: egg mass)				
Week 56–60	2.172	2.208	0.0160	0.14
Week 60–64	2.224	2.295	0.0165	0.02
Week 64–68	2.312	2.432	0.0248	0.01
Week 68–72	2.406	2.478	0.0359	0.19
Week 56–72	2.209	2.256	0.0089	0.01
Laying rate (%)				
Week 56–60	95.9	94.7	0.65	0.18
Week 60–64	94.6	94.9	0.71	0.76
Week 64–68	91.9	91.7	0.31	0.59
Week 68–72	91.3	91.5	0.55	0.76
Week 56–72	93.6	93.5	0.27	0.86
Egg weight (g)				
Week 56–60	62.6	62.1	0.24	0.20
Week 60–64	63.2	62.7	0.25	0.19
Week 64–68	65.2	64.8	0.23	0.21
Week 68–72	65.5	65.0	0.25	0.22
Week 56–72	64.1	63.6	0.22	0.15
Egg mass (g/d)				
Week 56–60	60.0	58.8	0.60	0.17
Week 60–64	59.8	59.5	0.63	0.76
Week 64–68	60.0	59.4	0.26	0.16
Week 68–72	60.0	59.5	0.44	0.68
Week 56–72	60.0	59.5	0.33	0.30

CON: Control diet; DON diet: diet contaminated with 2.45 mg/kg DON; SEM: standard error of the mean; BW: body weight; FI: feed intake; FCR: feed conversion ratio.

### Egg quality

**Fig. 1:**
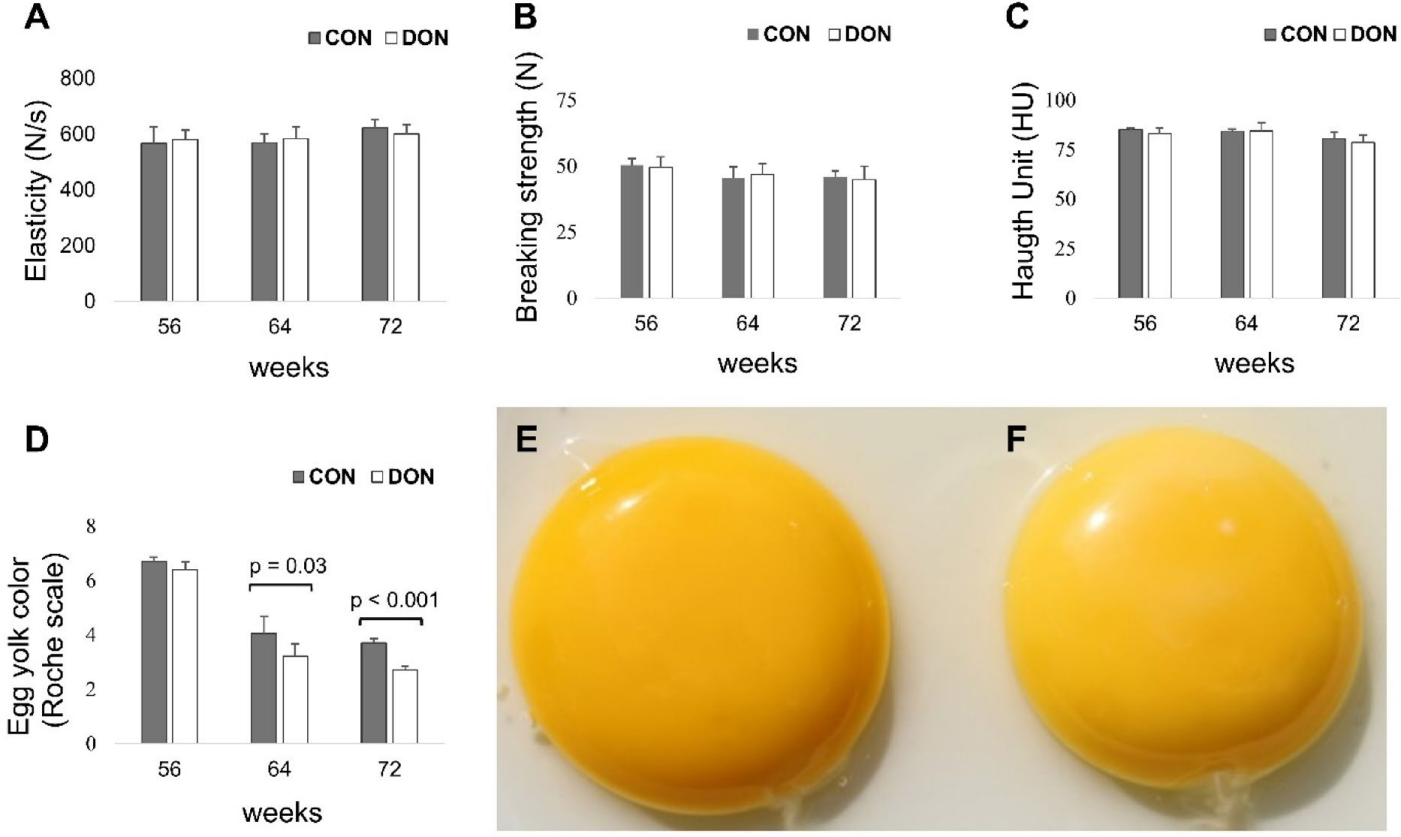
Mean (± SD) values of (**A**) elasticity (N/s), (**B**) breaking strength (N), (**C**) Haugh unit (HU), and (**D**) yolk colour (Roche scale). Panels (**E**) and (**F**) show representative egg yolks from laying hens fed the CON and DON-contaminated diets, respectively. Data were collected at the start of the experiment (56 weeks of age) and after 8 (64 weeks) and 16 weeks (72 weeks) of dietary exposure. CON: control diet; DON diet: diet contaminated with 2.45 mg/kg DON.

Egg quality was assessed based on eggshell elasticity, breaking strength, albumen thickness (Haugh unit), eggshell thickness, and yolk colour at 56 (start of the trial), 64, and 72 weeks of age. None of these parameters differed at the beginning of the trial. The dietary treatments had no effect on eggshell elasticity, breaking strength, or Haugh unit. However, yolk colour score significantly decreased in hens fed the DON diet, resulting in paler yolks at both 64 and 72 weeks of age (Fig. 1).

### Mean relative weight of the reproductive tract

Dietary exposure to DON did not affect the relative weights of the ovary, shell gland, or total reproductive tract, regardless of the exposure period (Table 4).

**Table 4:** Mean relative weight (% of BW) of reproductive tract of laying hens after 8 weeks (week 64) and 16 weeks (week 72) of dietary exposure.

	CON	DON	SEM	*p*-value
Week 64				
Ovary	3.33	3.26	0.16	0.75
Shell gland (full)	6.12	5.68	0.34	0.39
Shell gland (empty)	4.18	4.23	0.15	0.83
Reproductive tract	9.42	8.97	0.46	0.51
Week 72				
Ovary	3.96	3.72	0.29	0.57
Shell gland (full)	4.82	4.87	0.40	0.93
Shell gland (empty)	1.51	1.76	0.14	0.24
Reproductive tract	11.19	11.06	0.64	0.89

CON: Control diet; DON diet: diet contaminated with 2.45 mg/kg DON; SEM: standard error of the mean.

### Serum analysis

After 8 weeks of dietary exposure (64 weeks of age) to the naturally contaminated DON diet, serum levels of DON (*p* < 0.01) and Ca (*p* = 0.03) increased, while serum levels of P (*p* = 0.02), glucose (*p* < 0.01), and β-carotene (*p* < 0.001) decreased. In addition, the serum Ca: P ratio increased from 1.9 to 2.5 (*p* < 0.01) in hens fed the DON diet from 56 to 64 weeks of age. By 72 weeks of age, the differences were limited to higher serum DON levels (*p* = 0.04) and lower β-carotene concentrations (*p* < 0.01) compared with the CON group (Table 5).

**Table 5:** Serum biochemical parameters of laying hens fed control or DON-contaminated diets after 8 (week 64) and 16 weeks (week 72) of exposure.

	CON	DON	SEM	*p*-value
Week 64				
DON (ng/ml)	0.03	0.57	0.11	< 0.01
Ca (µmol/ml)	6.5	7.1	0.19	0.03
P (µmol/ml)	3.4	3.0	0.14	0.02
Ca: P	1.9	2.5	0.12	< 0.01
Glucose (µmol/ml)	17.4	14.6	0.65	< 0.01
Albumin (mg/ml)	21.3	20.2	0.52	0.60
Creatinine (nmol/ml)	9.0	8.8	0.40	0.77
CK (U/l)	2764	3186	372.4	0.44
ALT (ng/ml)	8.1	7.4	0.70	0.53
AST (ng/ml)	198	190	10.5	0.60
Lipase (U/l)	44.3	42.3	2.40	0.58
Bilirubin (nmol/ml)	0.15	0.16	0.05	0.87
CHOL (µmol/ml)	4.6	4.9	0.42	0.65
β-carotene (ng/ml)	43.6	19.5	4.1	< 0.001
Week 72				
DON (ng/ml)	0.03	0.35	0.10	0.04
Ca (µmol/ml)	6.6	6.7	0.19	0.68
P (µmol/ml)	2.9	2.9	0.22	1.00
Ca: P	2.4	2.4	0.16	0.70
Glucose (µmol/ml)	13.9	13.9	0.96	0.98
Albumin (mg/ml)	20.2	20.6	0.41	0.47
Creatinine (nmol/ml)	9.2	8.7	0.32	0.28
CK (U/l)	2614	2825	294.2	0.62
ALT (ng/ml)	5.0	5.0	0.66	0.96
AST (ng/ml)	203	209	11.9	0.76
Lipase (U/l)	47.0	46.5	1.98	0.86
Bilirubin (nmol/ml)	0.19	0.14	0.05	0.51
CHOL (µmol/ml)	4.5	4.3	0.37	0.75
β-carotene (ng/ml)	26.7	14.7	2.84	< 0.01

Analysed serum parameters with their respective limit of quantification: Deoxynivalenol (DON; 0.25 ng/ml), Calcium (Ca; 0.2 µmol/ml), Phosphorus (P; 0.1 µmol/ml), Glucose (0.94 µmol/ml), Albumin (3 mg/ml), Creatinine (15 nmol/ml), Creatine kinase (CK; 7 U/l), Alanine transaminase (ALT; 5 ng/ml), Aspartate transaminase (AST; 5 ng/ml), Lipase (3 U/l), Bilirubin (2.5 nmol/ml), Cholesterol (CHOL; 0.1 µmol/ml), β-Carotene (5 ng/ml).

CON: control diet; DON diet: diet contaminated with 2.45 mg/kg DON; SEM: standard error of the mean.

### Histological analysis

**Fig. 2:**
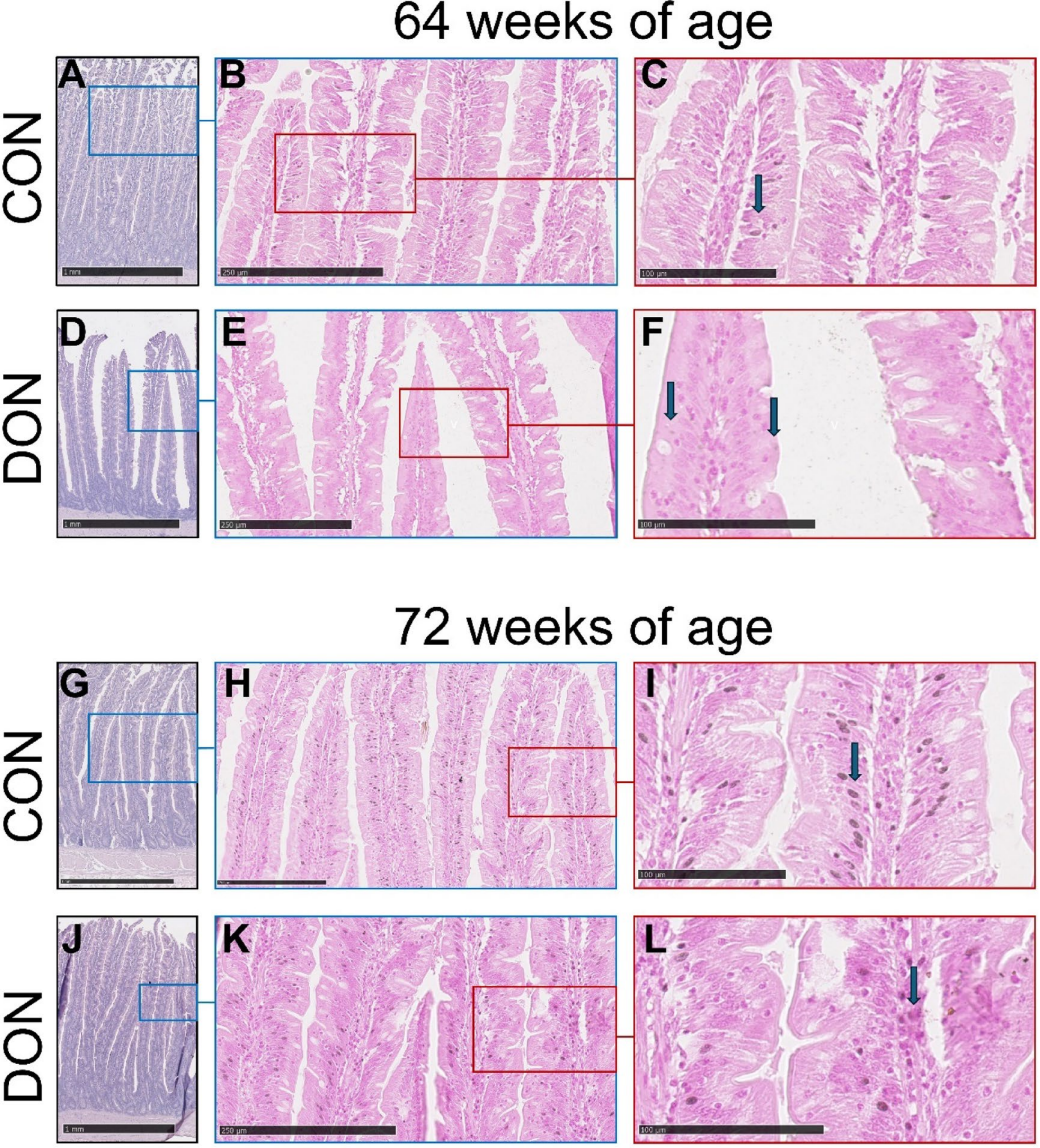
Representative images of haematoxylin–eosin (**A**, **D**, **G**, **J**) and von Kossa (**B**, **C**, **E**, **F**, **H**, **I**, **K**, **L**) staining of the duodenum from laying hens after 8 weeks (64 weeks of age) and 16 weeks (72 weeks of age) of dietary exposure. After 8 weeks of DON exposure (64 weeks; **D**), the contact between adjacent villi was reduced compared with the CON group (**A**). After 16 weeks of DON exposure (72 weeks; **G**, **J**), no noticeable differences were observed. Von Kossa staining was used to visualise Ca deposits, visible as dark precipitates (arrows). Scale bars: A, D, G, J = 1 mm; B, E, H, K = 250 μm; C, F, I, L = 100 μm. CON: control diet; DON diet: diet contaminated with 2.45 mg/kg DON.

Exposure to DON led to a decrease in villus height (*p* = 0.02) after 8 weeks of dietary exposure (64-week-old laying hens). After 16 weeks of exposure (72-week-old laying hens), the VH: CD ratio was reduced (*p* = 0.03) (Table 6). No other significant differences were observed. Figure 2 shows representative images of the duodenum from hens aged 64 and 72 weeks subjected to the dietary interventions. Notably, after 8 weeks of dietary DON exposure (64 weeks of age), the distance between villi increased, indicating reduced villus density (Fig. 2D); however, this effect was no longer evident after 16 weeks of exposure (72 weeks of age) (Fig. 2J). Cells containing Ca deposits are indicated in Fig. 2. It is noteworthy that these deposits were more numerous in the villi of 72-week-old hens, irrespective of the dietary intervention.

**Table 6:** Duodenal morphology and calcium staining parameters of laying hens fed control or DON-contaminated diets after 8 (week 64) and 16 weeks (week 72) of exposure.

	CON	DON	SEM	*p*-value
Week 64				
Villus height (µm)	1180	1007	46.30	0.02
Crypt depth (µm)	296	308	24.33	0.72
VH: CD	4.47	3.58	0.336	0.09
Villus area (mm^2^)	0.21	0.21	0.031	0.92
Damage score	1.69	2.04	0.258	0.36
Ca-positive cells/villus	14.38	9.15	4.062	0.38
Ca-positive cells/villus area	74.88	72.90	30.23	0.97
Week 72				
Villus height (µm)	1200	1072	53.88	0.12
Crypt depth (µm)	302	330	13.46	0.16
VH: CD	4.21	3.42	0.216	0.03
Villus area (mm^2^)	0.16	0.15	0.011	0.74
Damage score	1.18	1.18	0.122	1.00
Ca-positive cells/villus	40.25	16.08	9.78	0.11
Ca-positive cells/villus area	222.8	101.2	32.26	0.12

CON: Control diet; DON diet: diet contaminated with 2.45 mg/kg DON; SEM: standard error of the mean; VH: CD: villus height: crypt depth ratio.

**Fig. 3:**
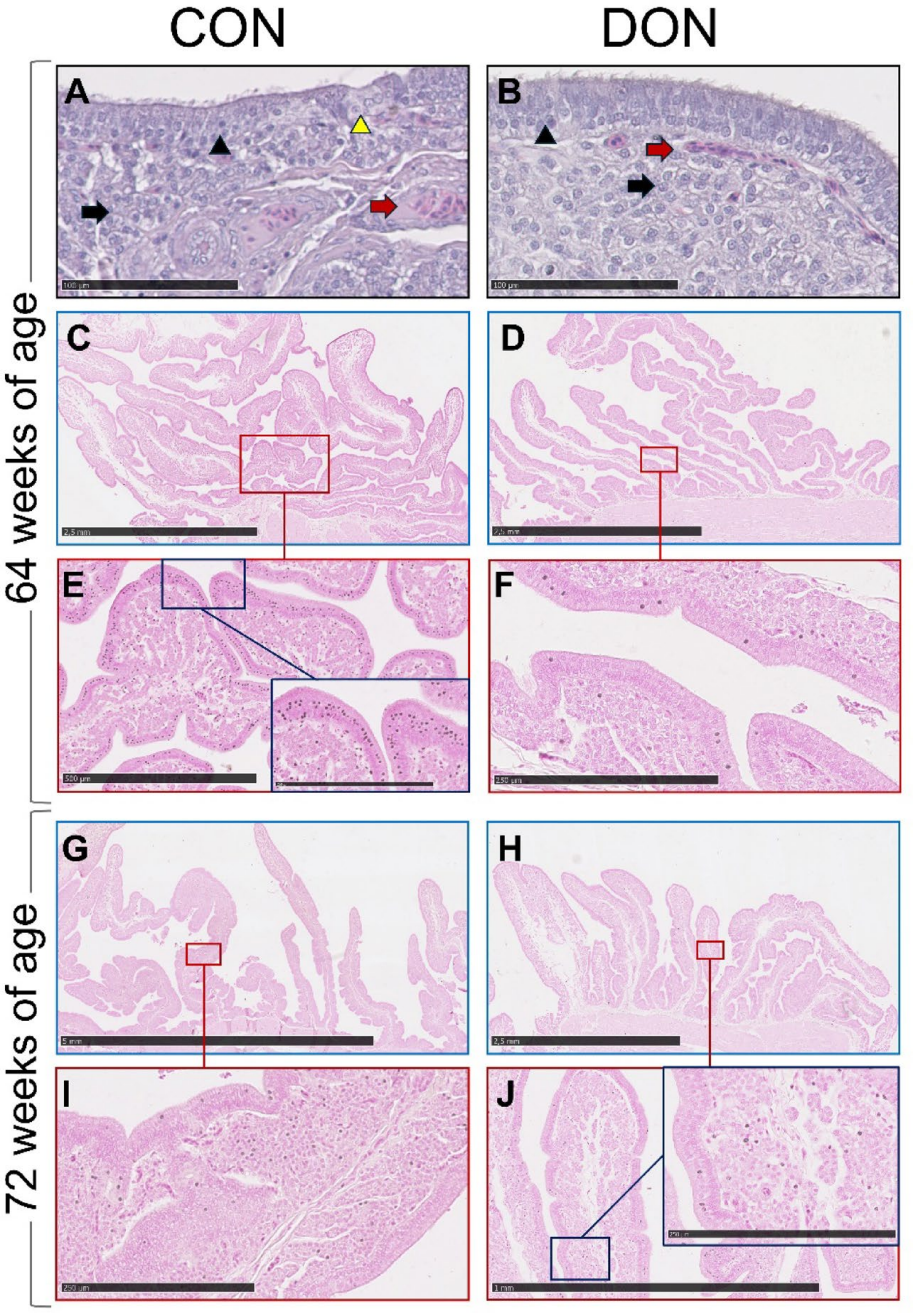
Representative images of the shell gland from laying hens subjected to 8 weeks (64 weeks of age) of dietary exposure following haematoxylin–eosin (**A**, **B**) and von Kossa (**C**–**F**) staining, and to 16 weeks (72 weeks of age) of dietary exposure following von Kossa staining (**G**–**J**). Mucosal folds exhibited similar height and area regardless of dietary treatment. After 8 weeks of DON exposure (**F**), the number of Ca-labelled cells per mucosal fold and per mm² of mucosal fold decreased compared with the CON group (**E**). This difference was not observed after 16 weeks of DON exposure (**I**, **J**). Von Kossa staining was used to visualise Ca deposits, visible as dark precipitates. Black arrows: tubular glands; red arrows: blood vessels; black arrowheads: ciliated epithelial cells; yellow arrowhead: non-ciliated epithelial cells. Scale bars: G = 5 mm; C, D, H = 2.5 mm; J = 1 mm; E = 500 μm; F, I = 250 μm; insets E, J = 250 μm; A, B = 100 μm. CON: control diet; DON diet: diet contaminated with 2.45 mg/kg DON.

Figure 3 shows histological sections of the shell gland, focusing on the mucosal folds. The treatments did not affect the integrity or morphology of the shell gland, which exhibited a normal, intact stratified cuboidal epithelium with both ciliated and non-ciliated cells, as well as tubular glands (Fig. 3A and B). However, differences were observed in the number of Ca-labelled cells per mucosal fold (Fig. 3C and D). Dietary exposure to DON did not influence the height or area of the mucosal folds in the shell gland. Nonetheless, the number of Ca-positive cells per mucosal fold (*p* < 0.01) and per mm^2^ of mucosal fold (*p* = 0.02) decreased after 8 weeks of dietary exposure (64 weeks of age), while no differences were observed after 16 weeks (72 weeks of age) (Table 7; Fig. 3E and F).

**Table 7:** Shell gland morphology and calcium staining parameters of laying hens fed control or DON-contaminated diets after 8 (week 64) and 16 weeks (week 72) of exposure.

	CON	DON	SEM	*p*-value
Week 64				
Tubular gland per mm	2.02	1.89	0.07	0.24
Tubular gland height (µm)	2180	2000	100.9	0.27
Tubular gland area (mm^2^)	0.95	0.81	0.09	0.33
Ca-positive cells/tubular gland	217	86	19.7	< 0.01
Ca-positive cells/tubular gland area	237	122	23.2	0.02
Week 72				
Tubular gland per mm	2.42	2.26	0.21	0.61
Tubular gland height (µm)	2414	2172	209.8	0.43
Tubular gland area (mm^2^)	0.96	0.78	0.10	0.22
Ca-positive cells/tubular gland	85.0	60.4	19.7	0.39
Ca-positive cells/tubular gland area	134	113	42.8	0.73

CON: Control diet; DON diet: diet contaminated with 2.45 mg/kg DON; SEM: standard error of the mean.

Regarding the ovarian population, 8 weeks of dietary exposure to DON (64 weeks of age) led to a reduction in the percentage of normal primordial (*p* = 0.02) and primary (*p* < 0.01) follicles. Similarly, after 16 weeks of exposure (72 weeks of age), the percentages of normal primordial (*p* < 0.001) and primary (*p* < 0.001) follicles, as well as the total number of large white follicles (*p* < 0.01), were significantly reduced (Table 8).

**Table 8:** Ovarian follicle morphology and follicular population of laying hens fed control or DON-contaminated diets after 8 (week 64) and 16 weeks (week 72) of exposure.

	CON	DON	SEM	*p*-value
Week 64				
Primordial follicles (% normal)	64.4	45.1	5.18	0.02
Primary follicles (% normal)	79.8	57.6	2.29	< 0.01
Secondary follicles (% normal)	85.4	78.2	2.73	0.09
Prehierarchical (% normal)	76.2	66.8	5.47	0.24
Prehierarchical (total number)				
Large white	22.2	20.3	2.30	0.57
Small yellow	9.8	7.4	0.86	0.08
Hierarchical (total number)	5.9	6.3	0.21	0.19
Week 72				
Primordial follicles (% normal)	61.7	27.5	3.26	< 0.001
Primary follicles (% normal)	74.8	57.4	2.51	< 0.001
Secondary follicles (% normal)	82.8	83.4	2.95	0.89
Prehierarchical (% normal)	74.6	78.3	3.18	0.42
Prehierarchical (total number)				
Large white	28.1	20.0	1.90	< 0.01
Small yellow	5.8	4.7	0.50	0.15
Hierarchical (total number)	5.6	5.3	0.30	0.37

CON: Control diet; DON diet: diet contaminated with 2.45 mg/kg DON; SEM: standard error of the mean.

**Fig. 4:**
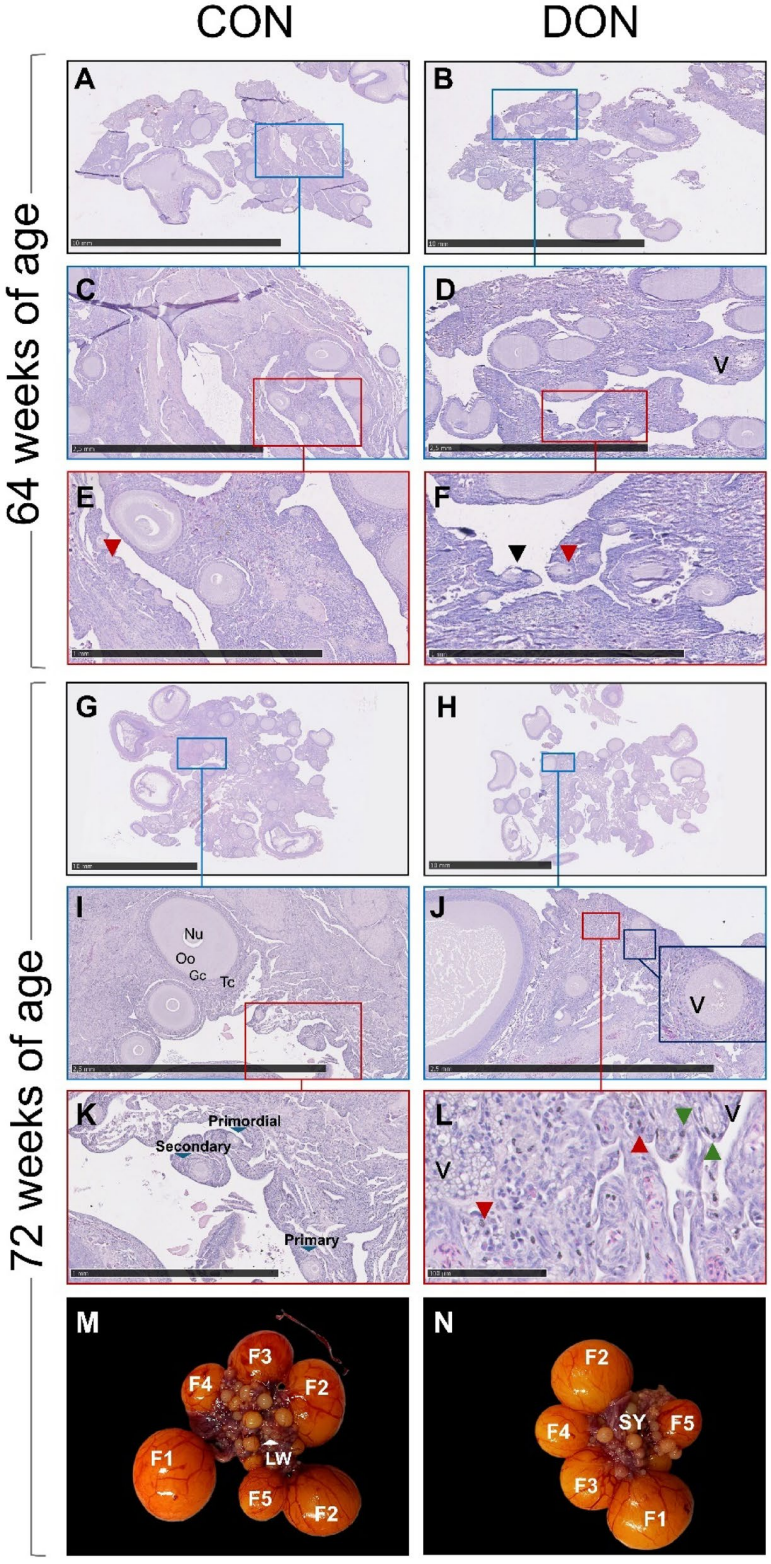
Representative images of haematoxylin–eosin staining of the ovary from laying hens after 8 (64 weeks of age) and 16 weeks (72 weeks of age) of dietary exposure (**A**–**L**), and photographs of ovaries from 72-week-old hens fed the experimental diets (**M**,** N**). Ovarian follicles are shown at different developmental stages: primordial follicles (oocytes surrounded by a single layer of flat pre-granulosa cells, representing the reserve pool of gametes), primary follicles (oocytes surrounded by one layer of cuboidal granulosa cells), and secondary follicles (oocytes surrounded by two or more layers of cuboidal granulosa cells, with larger follicles also encased by a theca layer). Follicles were classified as normal or degenerated. Degeneration was identified by nuclear aggregation and shrinkage, with or without cytoplasmic vacuolation. Normal primordial, primary, and secondary follicles are indicated by blue arrowheads (**K**), whereas atretic primordial follicles with pycnotic nuclei are marked with red arrowheads (**E**, **F**, **L**). Green arrowheads indicate pycnotic granulosa cells surrounding degenerated oocytes (**L**), and black arrowheads show oocytes detached from surrounding granulosa cells, another indicator of atresia. Degenerated follicles in the DON group also exhibited cytoplasmic vacuolation, marked by the letter ‘v’ (**D**, **J**, **L**). Photographs of ovaries from 72-week-old hens show large white (LW), small yellow (SY), and hierarchical follicles (F5–F1). The hierarchical follicles in the DON group (**N**) display a lighter orange colour compared with those in the CON group (**M**). Gc: granulosa cells; Nu: nucleus; Oo: oocyte; Tc: theca cells. Scale bars: A, B, G, H = 10 mm; C, D, I, J = 2.5 mm; E, F, K = 1 mm; **L** = 100 μm. CON: control diet; DON diet: diet contaminated with 2.45 mg/kg DON.

Figure 4 presents ovaries from 64- and 72-week-old laying hens fed either the CON or DON diets. Follicular atresia, characterised by oocytes with pycnotic nuclei, was observed in the ovaries of both CON (Fig. 4E) and DON (Fig. 4F and L) groups, though it was more frequent in primordial and primary follicles of hens fed the DON diet. Additionally, vacuolation of the oocyte cytoplasm and the presence of pycnotic bodies in the ovarian stroma and granulosa cells were observed in the ovaries of hens from the DON group (Fig. 4D, J, and L). It is worth noting that the hierarchical follicles in the DON group (Fig. 4N) displayed a lighter orange colour compared with those from the CON group (Fig. 4M).

### mRNA relative expression in the duodenum and shell gland

**Fig. 5:**
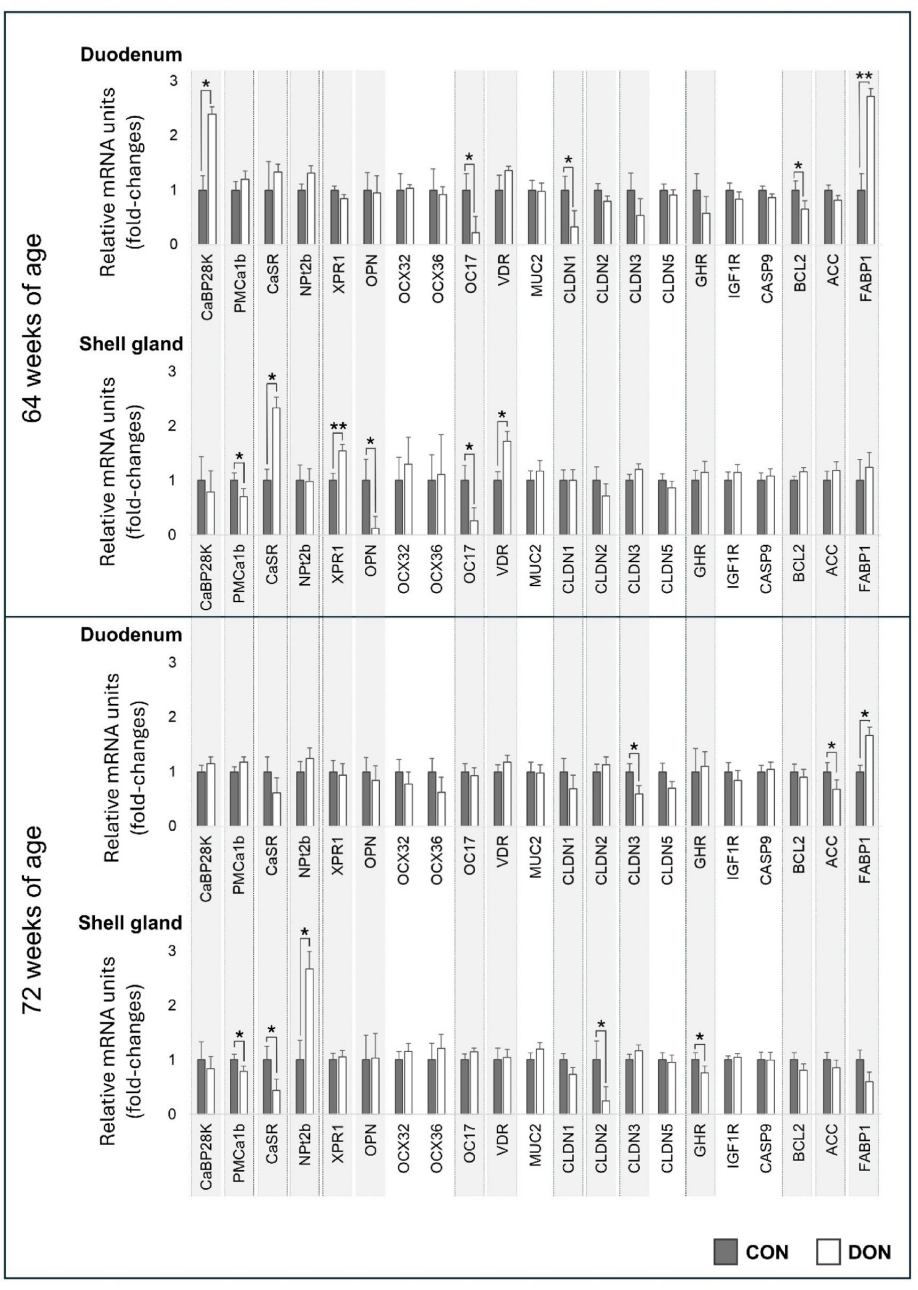
Mean (± SD) mRNA expression of target genes in the duodenum and shell gland of laying hens after 8 weeks (64 weeks of age) and 16 weeks (72 weeks of age) of dietary exposure. Data are expressed as fold-change relative to the respective CON group. CON: control diet; DON diet: diet contaminated with 2.45 mg/kg DON.

Figure 5 summarises the relative mRNA expression of the selected target genes analysed in the duodenum and shell gland of the laying hens. Regardless of tissue type or exposure duration, no differences were observed in the relative expression of *OCX32*, *OCX36*, *MUC2*, *CLDN5*, IGF 1 receptor (*IGF1R*), or *CASP9*. After 8 weeks of DON exposure (64-week-old hens), the duodenum showed upregulation of *CaBP28k* (*p* = 0.03) and *FABP1* (*p* = 0.01), alongside downregulation of *OC17* (*p* = 0.03), *CLDN1* (*p* < 0.05), and *BCL2* (*p* < 0.05). In the shell gland, DON exposure over the same period (64 weeks of age) led to upregulation of *CaSR* (*p* = 0.03), *XPR1* (*p* < 0.01), and *VDR* (*p* = 0.03) and downregulation of *PMCA1b* (*p* = 0.001), *OPN* (*p* < 0.05), and *OC17* (*p* = 0.04).

After 16 weeks of DON exposure (72-week-old hens), the duodenum exhibited upregulation of *FABP1* (*p* = 0.04) and downregulation of *CLDN3 (p* = 0.02) and *ACC* (*p* = 0.04). In the shell gland, DON exposure for 16 weeks resulted in upregulation of *NPt2b* (*p* < 0.05) and downregulation of *PMCA1b* (*p* = 0.03), *CaSR* (*p* = 0.04), *CLDN2* (*p* < 0.05), and *GHR* (*p* = 0.04).

### Excreta metabolomics

In total, 1040 metabolites were identified in the excreta of hens from the CON and DON groups across both sampling periods (64 and 72 weeks of age). After filtering based on the interquartile range, 624 metabolites remained for analysis.

**Fig. 6:**
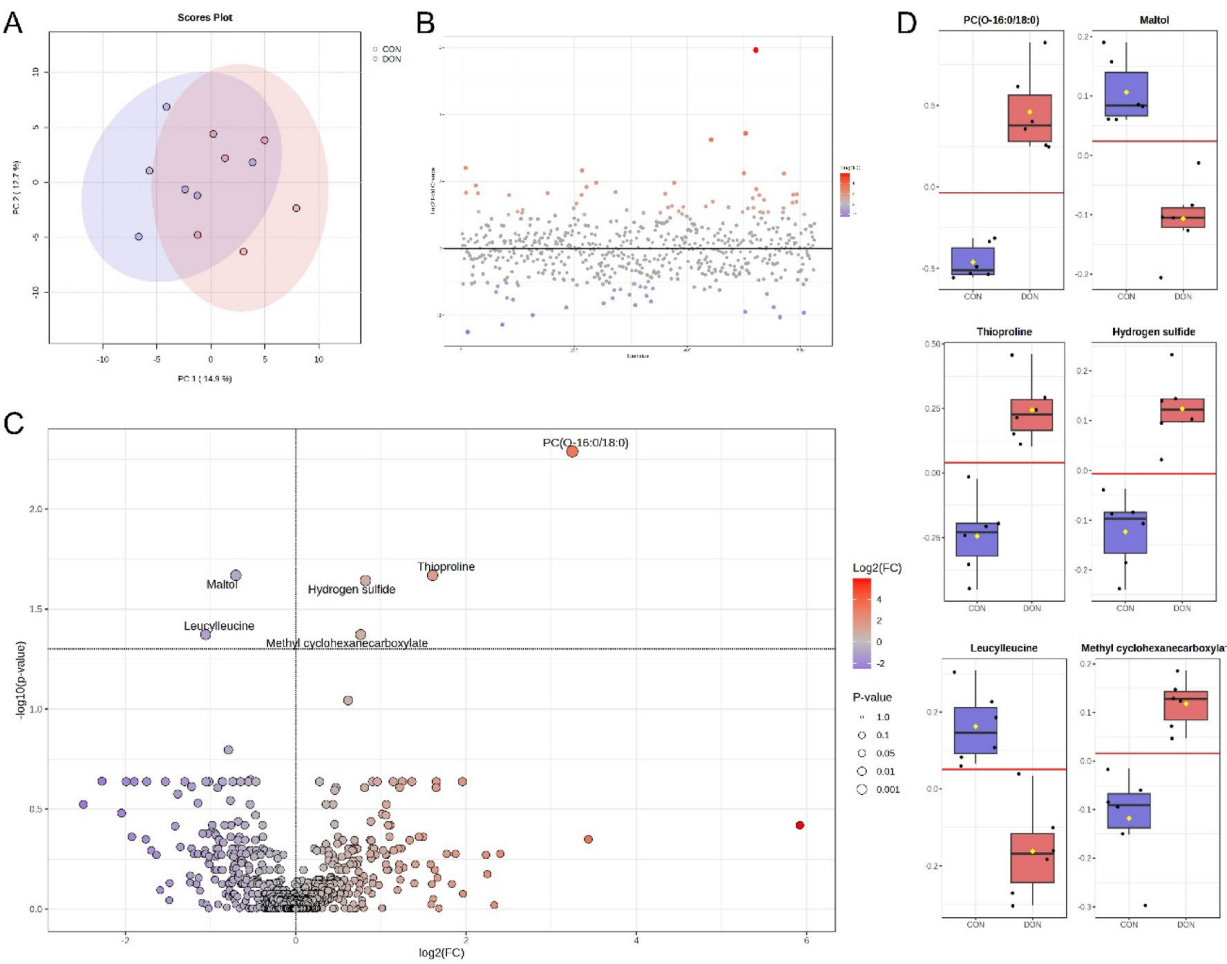
Univariate analysis of excreta metabolites from laying hens. (**A**) PCA scores plot; (**B**) fold-change analysis showing metabolite features contributing to separation of metabolomic profiles; (**C**) volcano plot comparing CON and DON datasets, with significant up- and down-regulated features identified by molecular name; (**D**) box plots showing the distribution of individual metabolites between CON and DON groups (*p* < 0.001; q < 0.05). CON: control diet; DON diet: diet contaminated with 2.45 mg/kg DON.

No significant differences were detected, and no clear separation between clusters was observed in the principal component analysis (PCA) of excreta samples collected after 8 weeks of dietary exposure (64-week-old hens). After 16 weeks of exposure (72-week-old hens), unsupervised PCA analysis showed some overlap between the CON and DON groups (Fig. 6A). Fold-change analysis identified groups of metabolite features contributing to the separation of metabolomic profiles (Fig. 6B). Among these, six metabolites—PC(O-16:0/18:0), thioproline, H_2_S, maltol, leucylleucine, and methyl cyclohexanecarboxylate—were found to differ significantly between treatments (*p* < 0.05; q < 0.05), as shown in the volcano plot (Fig. 6C). According to receiver operating characteristic (ROC) analysis, all six biomarkers exhibited an area under the curve (AUC) of 1.000, with both sensitivity and specificity values of 1.000 (Fig. 6D; Table 9).

Although DON itself was not detected in the excreta, its metabolite deoxynivalenol-3-sulphate (DON-3 S) was observed. The peak areas of DON-3 S were significantly higher in the DON groups than in the CON groups at both sampling points (*p* < 0.001). Specifically, the peak areas of DON-3 S were 0.36 and 0.32 in the CON groups after 8 and 16 weeks of exposure (64- and 72-week-old hens, respectively), whereas they were 2.67 and 2.39 in the DON groups at the same time points.

**Table 9:** Receiver operating characteristic analysis of potential metabolite biomarkers in excreta from 72-week-old laying hens fed CON or DON-contaminated diets.

CON versus DON	AUC (95% CI)	Sensitivity	Specificity	Threshold
Hydrogen sulphide	1.000 (1.000)	1.000	1.000	0.007
Leucylleucine	1.000 (1.000)	1.000	1.000	0.050
Maltol	1.000 (1.000)	1.000	1.000	0.024
Methyl cyclohexanecarboxylate	1.000 (1.000)	1.000	1.000	0.016
PC (O-16:0/18:0)	1.000 (1.000)	1.000	1.000	0.033
Thioproline	1.000 (1.000)	1.000	1.000	0.040

CON: Control diet; DON diet: diet contaminated with 2.45 mg/kg DON; AUC: area under the curve; CI: confidence interval; PC: phosphatidylcholine.

## Discussion

The findings of the present study demonstrate that dietary exposure to 2.45 mg/kg DON negatively affected intestinal health, reproductive function, and production performance in laying hens. After 8 weeks of exposure (64-week-old hens), impairment in the FCR was already evident. This negative effect persisted after 12 weeks (68-week-old hens); however, by 16 weeks (72-week-old hens), no significant differences were detected between experimental groups. Previous studies have also reported adverse effects of high DON levels (12.1 mg/kg), showing increased FCR after 8 and 12 weeks of exposure^21^, as well as of 2 mg/kg DON in combination with extremely high levels of aflatoxin B1 (2 mg/kg), which resulted in increased FCR after 6 and 10 weeks^22^. In the current trial, the lack of differences after prolonged exposure may have been age-related because the hens were already in the natural decline phase of egg production, potentially masking treatment effects. Egg production reaches a peak when laying hens are around 30 weeks of age, and this production begins to decline after 40–45 weeks of age, with a more significant decrease, referred as mid-decline, occurring after 67 weeks of age^23^. Alternatively, it may reflect physiological adaptation to the contaminated diet, allowing the birds to maintain egg production at the cost of less efficient energy utilisation.

Analysis of the physical characteristics of the eggshell revealed no dietary effects; however, yolk colour was significantly paler in hens fed the DON diet. The paler yolks observed in this study cannot be directly attributed to DON impairing carotenoid absorption. They are more likely due to differences in the dietary content of these pigments because the DON diet contained lower levels of lutein, xanthophylls, and zeaxanthin than the CON diet. The CON and DON diets were prepared using two different maize batches. This reduced pigment content may reflect natural variation in carotenoid levels among maize sources^24^ or may result from the plant’s defensive response to *Fusarium* infection, during which carotenoids are utilised to counteract fungal invasion^25^, leading to their increased degradation^26^. In the present study, serum β-carotene concentrations were lower in the DON group and declined over time, corresponding with the observed decrease in yolk pigmentation. However, because both diets contained β-carotene below the quantification limit, a direct dietary–serum correlation could not be established.

Serum analysis confirmed DON exposure and revealed a significant decrease in circulating glucose and P levels. A DON-induced reduction in glucose absorption has previously been demonstrated in vitro using intestinal segments from 72-week-old laying hens^27^. In broiler chickens, a linear decline in serum P and bone mineralisation was reported as dietary DON levels increased from 2.5 to 10 mg/kg^28^. Those authors attributed the effect to both decreased FI and disrupted mineral homeostasis. Although serum Ca levels were unaffected at the highest DON doses, Ca concentrations in the femur and tibiotarsus were significantly reduced. Unfortunately, bone Ca content was not assessed in the present trial.

Unlike the findings in broilers^28^, serum Ca levels in this study did not remain similar to the CON group. Instead, they increased significantly after 8 weeks of DON exposure (64-week-old hens) and returned to levels comparable with the CON group after 16 weeks (72-week-old hens). The apoptotic and cell-cycle arrest effects of DON on intestinal cells^29^ were reflected in decreased VH and VH: CD ratios, along with BCL2 downregulation in the duodenum. These alterations would normally suggest reduced nutrient absorption capacity; however, DON exposure led to increased Ca absorption, as indicated by serum levels. Histological staining for Ca-positive cells in the duodenum showed no differences between groups, but mRNA expression analysis revealed upregulation of *CaBP28k* after 8 weeks (64-week-old hens), suggesting enhanced Ca transport from the apical to the basolateral membrane^30^. This transient increase may represent a compensatory mechanism to preserve Ca homeostasis.

Support for this compensatory response comes from the concurrent upregulation of *FABP1*, likely reflecting an attempt to increase fatty acid uptake into intestinal cells. After 8 weeks of exposure, *FABP1* expression in the DON group was roughly threefold higher than in the CON group, and after 16 weeks, about twofold higher, coinciding with a downregulation of *ACC*. This pattern suggests that the duodenum’s adaptive capacity to DON exposure was reaching its limit.

The decreased expression of *OC17* in the duodenum, accompanied by its downregulation in the shell gland, may indicate compromised eggshell calcification. OC17 also exhibits antimicrobial activity and is considered a potential marker of the health status of fertilised eggs^31^. At the molecular level of the shell gland, both *OPN* and *PMCA1b* were downregulated after 8 weeks of DON exposure, in addition to *OC17*. While OC17 contributes to the initiation of eggshell mineralisation, OPN regulates crystal orientation during rapid calcification, influencing shell hardness^31^. To counteract these effects, genes involved in Ca mobilisation (*CaSR*), vitamin D response (*VDR*), and P transport (*XPR1*) were upregulated.

The downregulation of *PMCA1b* may have reduced Ca transport from the shell gland for calcium carbonate deposition in the shell. Consistently, the number of Ca-positive cells in the shell gland decreased significantly after 8 weeks of DON exposure. Although these differences were no longer evident after 16 weeks (72-week-old hens), *PMCA1b* expression remained downregulated. While no visible changes in eggshell physical characteristics were detected, these molecular alterations could impair eggshell quality in the long term. For instance, the early upregulation of *CaSR* was followed by its downregulation after 16 weeks, along with reduced *GHR* expression—an indication of declining Ca mobilisation and impaired shell formation^32^. The upregulation of *NPt2b* suggests an adaptive response to reduced P availability^33^.

Although downregulation of *CLDN* genes was observed in both the duodenum and shell gland, these changes were limited to individual tight-junction proteins, while others remained unaffected. Therefore, based on the present data, increased tissue permeability cannot be inferred.

The negative effect of DON also influenced the population of ovarian follicles, including the large white follicles, but most notably the ovarian reserve—namely, the primordial and primary follicles, which presented not only pyknotic nuclei but had cytoplasm vacuolation as one of the main alterations. This effect of DON has previously been demonstrated in mammals with a diminished population of ovarian preantral follicles (primordial, primary, and secondary)^34^ and follicular atresia characterized by apoptosis of ovarian stroma, oocytes, and granulosa cells^18^. It was previously described that *Fusarium* toxins induce vacuolation in the cytoplasm of oocytes, an apoptosis-independent cell-death pathway^35^. Particularly DON exposure resulted in the cytoplasm vacuolation of oocytes^14^ and of somatic cells^36^.

Because ovarian follicles are non-renewable and represent the reserve pool of gametes, a long-term decline in egg production can reasonably be anticipated.

Droppings were collected from the pens for metabolomic analysis to identify potential biomarkers related to intestinal health and the impact of inefficient nutrient utilisation. No differences were observed in samples collected after 8 weeks of exposure; however, after 16 weeks, six untargeted metabolites were found to be regulated by DON exposure. Unfortunately, there is a lack of research on the presence of these metabolites in poultry excreta because most existing studies focus on humans.

Maltol, a natural compound linked to intestinal immune and anti-inflammatory responses and produced by beneficial gut microbes^37^, was decreased in the excreta. This reduction may indicate diminished microbial synthesis or increased utilisation to counteract intestinal inflammation. Similarly, the decreased excretion of leucylleucine (Leu-Leu) suggests higher physiological demand to mitigate intestinal apoptosis and promote cell proliferation^38^. This may partly explain the recovery in villus height observed at the end of the trial compared with week 8 (64-week-old hens).

The increased excretion of phosphatidylcholine (PC)(O-16:0/18:0), a membrane lipid, suggests disrupted lipid metabolism and has been associated with intestinal inflammation in humans^39^. This finding is further supported by the concurrent rise in methyl cyclohexanecarboxylate, which is also found at elevated levels in the excreta of patients with irritable bowel syndrome^40^. Elevated thioproline levels indicate cellular stress, reflecting its detoxifying role; after trapping nitrite, it is subsequently excreted^41^.

Intestinal stress may lead to inefficient nutrient utilisation, particularly of amino acids and proteins. As a consequence, increased fermentation of sulphur-containing amino acids and proteins can elevate the excretion of H_2_S^42^—a volatile sulphur compound that, along with ammonia and carbon dioxide, poses health risks to both poultry and humans, as well as to the environment. A limitation of this study was the inability to measure on-site gas emissions to correlate with the metabolomic data. Conventional continuous monitoring is characterised by measuring gas emission in the entire house rather than per individual pen. Therefore, it was not possible to confirm the environmental impact of DON during the present trial, where all the experimental pens were located in the same barn. Future trials should incorporate chamber methods to estimate gas emission related to dietary DON exposure.

In conclusion, this study demonstrated that dietary exposure to DON at 2.45 mg/kg—approximately half of the maximum level recommended by the European Food Safety Authority (5.0 mg/kg)^2^—can reduce feed efficiency in laying hens after short-term exposure. The absence of performance differences between treatments after 16 weeks (72-week-old hens) may be linked to the birds’ age because they were already experiencing a natural decline in egg production. Therefore, further research using younger hens at peak production is warranted. Particular attention should also be given to breeder flocks because fertilised eggs from DON-exposed hens may yield weaker embryos and chicks. Although the physical characteristics of the eggshell were not altered by this transient exposure, imbalances in Ca and P metabolism at the molecular level were evident. Ovarian function was also compromised due to degeneration of the follicular reserve pool. Importantly, replacing the contaminated diet with a higher-quality one would not restore the depleted ovarian follicle population, leading to an earlier decline in egg production.

## Methods

### Ethics statement

The animal trial was approved by the Dutch Central Authority for Scientific Procedures on Animals (project license AVD24600202114740) and Animal Care and Use Committee of Schothorst Feed Research (SFR; Lelystad, the Netherlands), and conducted at the research facility of SFR. The protocol of the experiment was carried out in compliance with the ARRIVE guidelines and in accordance with the Dutch law on animal experimentation, which complies with the European Directive 2010/63/EU on the protection of animals used for scientific purposes.

### Laying hens and housing

At 53 weeks of age, 600 healthy Dekalb White laying hens were transferred from a commercial aviary facility to the experimental facilities of Schothorst Feed Research (Lelystad, the Netherlands). The laying hens had similar weight and production performance before starting the trial, with an average BW of 1676 g (± 17 g), an average laying percentage of 93% (± 0.3%), and an average FCR of 2.216 (± 0.016), and were randomly assigned to 12 floor pens (13.5 m^2^ each), with 50 hens per pen. Each pen was equipped with perches (approximately 18 cm/hen), two metal feeding bins (approximately 5 cm/hen feeder space), and a round drinker. Feed and water were provided *ad libitum*, and fresh wood shavings were used as bedding material. The facility was equipped with programmable artificial lighting, automated central heating, and forced ventilation. The lighting schedule was set to 14 h of light and 10 h of darkness per day, and the target room temperature was maintained at 20 °C ± 2°C. During the first 4 weeks (adaptation period), all hens were fed a control diet. The experimental period began at 56 weeks of age, when hens were fed the experimental diets for 16 weeks, until 72 weeks of age. Hens were inspected at least once daily by an animal caretaker, and any observations regarding health, behaviour, or mortality were recorded. No vaccinations were administered during the experiment.

### Experimental design

The experiment followed a completely randomised block design with two dietary treatment groups and six replicate pens per treatment. Laying hens in the CON group received a diet containing negligible levels of mycotoxins (see Table 1), while those in the DON group were fed a diet containing 2.45 mg/kg DON. Both diets were formulated to meet the nutritional requirements for laying hens as recommended by the Nutrient Requirements of Poultry^43^, and no colouring agents were added. The diets were analysed for β-carotene, lutein, and zeaxanthin concentrations using high performance liquid chromatography (HPLC) (Agilent, model 1260 Infinity, Agilent Technologies, Santa Clara, CA, USA). The xanthophyll concentration in the diets was measured at 474 nm using a spectrophotometer (Lambda^®^ 365+, Perkin Elmer, Waltham, MA, USA). All these carotenoids-related analyses were performed at (NutriControl B.V., Veghel, the Netherlands). Because the maize batches used in the trial differed in nutrient composition, the diets were adjusted to be isocaloric and isonitrogenous (Supplementary Table 1). Mycotoxin analysis was conducted by an independent accredited laboratory (BELAC 057-TEST/ISO17025; Primoris Holding, Ghent, Belgium) using liquid chromatography coupled with tandem mass spectrometry (LC–MS/MS).

### Measurements and sampling

To prevent biased observations or outcomes, the identification of the treatments was not disclosed to individuals participating in trial activities, including daily animal care, health assessment, weighing of feed and animals, and veterinary inspections. The individuals conducting the analysis only had access to the follow-up number of the pen and were not aware of the dietary treatment assigned to it.

BW, FI, laying rate, FCR, egg weight, and egg mass were recorded weekly per pen.

Eggshell breaking strength, elasticity, albumen thickness (Haugh unit), eggshell thickness, and yolk colour were measured using 30 eggs per pen at 56, 64, and 72 weeks of age by a specialised institute (Instituut voor Kwaliteitsbepalingen van Eieren, IKE, Amersfoort, the Netherlands).

At 64 and 72 weeks of age, two hens per pen, i.e., 12 hens per treatment per time point, were randomly selected and euthanised with carbon dioxide followed by exsanguination for blood and tissue collection. The BW of each sampled hen was recorded. Serum was obtained from approximately 5 ml of blood per hen by centrifugation (15 min at 1500 × *g*) and stored at − 20 °C for biochemical analysis. Duodenum samples were collected from two birds per pen after 8 and 16 weeks of dietary exposure (64 and 72 weeks of age, respectively). One segment was fixed in buffered formalin for histological analysis, and another was preserved in RNALater for mRNA expression analysis. The complete reproductive tract, including the ovary and shell gland, was also collected from the same hens. The weights of the complete reproductive tract, ovary, and shell gland (both full and empty) were recorded. The number of ovarian follicles measuring ≥ 1 mm was counted and classified as previously described^44^. Additionally, sections of the ovary and shell gland (~ 2 cm) were fixed in formalin for histological analysis, and another shell gland section (~ 2 cm) was preserved in RNALater for mRNA expression analysis. For excreta metabolomic analysis, fresh droppings were collected per pen after 8 and 16 weeks of dietary exposure (64 and 72 weeks of age, respectively). To avoid contamination with litter material, monitoring of the birds was performed to collect the samples immediately post-defecation. Samples were then pooled per pen, i.e., six samples per treatment and time point, and stored at − 80 °C until analysis.

### Serum analysis

Serum samples collected after 8 and 16 weeks of dietary exposure (i.e., at 64 and 72 weeks of age, respectively). The levels of DON, Ca, P, creatine kinase, creatinine, glucose, alanine transaminase, aspartate transaminase, lipase, bilirubin, cholesterol, and albumin were analysed with a high-throughput automated system (Cobas 8000 modular analyzer series, Roche Diagnostics International Ltd, Rotkreuz, Switzerland). β-carotene levels were analysed with HPLC (Shimadzu LC-20 series, Shimadzu Corporation, Kyoto, Japan). All these analyses were performed at Laboklin N.V. (Hoensbroek, the Netherlands).

### Histological analysis

Histological sections of the duodenum, shell gland, and ovaries were stained using the periodic acid–Schiff method with haematoxylin counterstaining. In addition, sections of the duodenum and shell gland were stained with von Kossa’s silver nitrate method for histochemical detection of Ca. All whole-slide images were acquired using the NanoZoomer-XR digital slide scanner (Hamamatsu Photonics K.K., Hamamatsu, Japan), visualised using NanoZoomer Digital Pathology view software (NDP.view2; Version 2.27.25), and analysed using NDP.analyze (Hamamatsu Photonics K.K., Hamamatsu, Japan; https://www.hamamatsu.com). For the duodenum, VH, CD, and villus area (VA) were measured in at least 10 villi per sample, and the VH: CD ratio was calculated^45^. For the shell gland, the height and area of the mucosal folds were measured, and the number of mucosal folds per millimetre of muscular layer was recorded. The number of Ca-positive cells was quantified and expressed per villus (duodenum) or per mucosal fold (shell gland), as well as per unit area of each tissue.

Each collected ovary was weighed, and the prehierarchical (> 1–8 mm) and hierarchical follicles were counted according to size: F5 (> 8–15 mm), F4 (> 15–20 mm), F3 (> 20–25 mm), F2 (> 25–30 mm), and F1 (> 30–35 mm)^44^. The prehierarchical follicles were further divided into large white (> 1–4 mm) and small yellow (> 5–8 mm) categories. The area of ovarian tissue prepared for histological analysis was measured, and follicles smaller than 1 mm were counted and classified. Ovarian follicles were categorised as follows: primordial follicles (an oocyte surrounded by a layer of flat pre-granulosa cells; up to 80 μm in diameter), primary follicles (an oocyte surrounded by a single layer of cuboidal granulosa cells; 80–300 μm), and secondary follicles (an oocyte surrounded by two or more layers of cuboidal granulosa cells, with the larger follicles also surrounded by a theca layer; 0.3–1 mm). Follicles were classified as either normal or degenerated. A follicle was considered degenerated if it showed nuclear aggregation and shrinkage, with or without detachment of the oocyte from the granulosa cells, or exhibited vacuolation of the oocyte cytoplasm^46^.

### mRNA relative expression by qRT-PCR

Samples of duodenum and shell gland were collected for the analysis of mRNA expression of markers related to their respective functions. RNA was extracted using the TRIzol method (TRI Reagent, Sigma Aldrich, St. Louis, MO, USA) following the manufacturer’s instructions. Reverse transcription was performed using the RevertAid First Strand cDNA Synthesis Kit (Thermo Fisher Scientific, Waltham, MA, USA). Primers (listed in Supplementary Table 2) were synthesised by Sigma Aldrich (Merck, Darmstadt, Germany). Primer specificity and efficiency were verified by qPCR using dilution series of pooled cDNA and a temperature gradient (55–65 °C) for primer annealing, followed by melting curve analysis. qPCR was performed using the Gentier 96E/96R real-time PCR detection system (Tianlong Science and Technology, Xi’an, Shaanxi, China). Data were analysed using the efficiency-corrected ΔΔCt method^47^. Fold-change values for genes of interest were normalised to the geometric mean of two housekeeping genes: *HPRT* (hypoxanthine-guanine phosphoribosyl transferase) and *GAPDH* (glyceraldehyde-3-phosphate dehydrogenase). The analysed genes included those involved in Ca and P transport (*CaBP28k*, *PMCA1b*, *CaSR*, *NPt2b*, *XPR1*), non-collagen proteins (*OPN* and *OC17*), eggshell calcification (*OCX32* and *OCX36*), tight junction integrity (*CLDN1*, *CLDN3*, *CLDN5*), mucin production (*MUC2*), growth regulation (*GHR* and *IGF1R*), fatty acid metabolism (*FABP1* and *ACC*), and apoptosis (*CASP9* and *BCL2*).

### Excreta metabolomic analyses

For metabolomic analysis, 200 mg of excreta were weighed into 2-ml microreaction tubes and dried for 24 h in a vacuum concentrator (Labconco, MO, USA). After dehydration, the samples were reweighed. Water was then added at a ratio of 40 µl per 20 mg sample, followed by vortex mixing. Metabolites were extracted by adding 160 µl of solvent mixture (ACN/water/formic acid, 49.5/49.5/1, v/v/v) per 20 mg sample, with vigorous vortexing and ultrasonication in an ice bath for 15 min. The samples were stored at − 20 °C overnight to allow protein precipitation, then centrifuged at 18,000 × *g* for 10 min at 4 °C. The supernatants were transferred to fresh tubes and diluted 1:10 with the same solvent mixture. Diluted extracts were filtered through PTFE membrane filters and placed into amber LC vials, and 5-µl aliquots were injected into the UHPLC–Q-TOF–MS system for analysis.

Samples were analysed using a UHPLC–Q-TOF–MS system. Analyses were performed on an Agilent 1290 Infinity II UHPLC system (Agilent Technologies) equipped with an autosampler, binary pump, and vacuum degasser, coupled to an Agilent 6546 LC/Q-TOF mass spectrometer (Santa Clara, CA, USA) operated in both positive and negative electrospray ionisation modes. A 5-µl injection volume was used, with a total run time of 25 min per analysis. Chromatographic separation was achieved on an Agilent Zorbax RRHD SB-C18 column (2.1 × 50 mm, 1.8 μm). The mobile phases consisted of Milli-Q water (A) and acetonitrile (B), both containing 0.1% formic acid. The gradient elution programme was as follows: 2% B at 0 min, ramped to 95% B at 22 min, returned to 5% B at 25 min, followed by a 3 min re-equilibration between injections. The flow rate was set at 0.4 ml/min. Source parameters for the Dual AJS ESI were as follows: drying gas temperature 325 °C, drying gas flow 10 l/min, nebuliser pressure 40 psig, sheath gas temperature 295 °C, sheath gas flow 12 l/min, capillary voltage 4000 V, nozzle voltage 500 V, fragmentor voltage 120 V, and skimmer voltage 70 V. Data were acquired in the m/z range of 100–1500 Da using a full-scan rate of 5 spectra/s and MS/MS acquisition at 3 spectra/s, allowing a maximum of two precursors per cycle, with collision energies set at 10, 20, and 40 eV.

An untargeted LC/Q-TOF metabolomics workflow was applied to characterise the metabolites present in the excreta samples. Data processing, chromatographic peak integration, and metabolite identification were performed using MassHunter Qualitative Analysis Software (Version B.08.00) and Personal Compound Database and Library Manager (Metabolomics PCDL; Version B.08.00) (Agilent Technologies, Santa Clara, CA, USA; https://www.agilent.com). Metabolite identification criteria included a minimum score threshold of 95% and a mass accuracy tolerance of 1 ppm.

### Data processing and statistical analyses

The pen was considered the experimental unit for all analyses. Data were analysed using analysis of variance using GenStat (Version 23.0, 2023; VSN International, Hemel Hempstead, UK; https://www.vsni.co.uk/software/genstat), and treatment means were compared using Tukey’s post hoc test. Differences were considered statistically significant at *p* ≤ 0.05. For metabolomics, data were log-transformed and mean-centred before being analysed by PCA to visualise variations and general clustering between groups^48^. To identify potential biomarkers of chronic DON exposure, univariate ROC analysis was performed to determine the AUC and assess the predictive performance of metabolites of interest, including accuracy (sensitivity and specificity), using an AUC of > 0.7 as the selection threshold^49^. Metabolomics data were further analysed using two-tailed Student’s *t*-tests to determine statistical significance (*p*-value) and false discovery rates (q-value). PCA and ROC curve analyses were conducted using MetaboAnalyst 6.0 software (Wishart Research Group, Edmonton, Canada; https://www.metaboanalyst.ca).

## Supplementary Information

Below is the link to the electronic supplementary material.

Supplementary Material 1

Supplementary Material 2

## Data Availability

The datasets generated during the current study are available from the corresponding author on reasonable request.
