# Supplementary material for: Short-term dietary deoxynivalenol exposure negatively affects performance, intestinal and reproductive functions in laying hens
Source: Sci Rep. 2026 Apr 11;16:16920. doi: 10.1038/s41598-026-46100-0 (PMC13230629; doi:10.1038/s41598-026-46100-0)
Supplement: Supplementary file 1 — Supplementary Material 1 [file 41598_2026_46100_MOESM1_ESM.docx]

**Supplementary table 1.** Composition and calculated nutrient content of the diets

| **Ingredient (%)** | **CON** | **DON** |
| --- | --- | --- |
| Corn | 60.00 | 60.00 |
| Sunflower meal | 10.00 | 8.76 |
| Soybean meal | 8.36 | 11.60 |
| Rape seed meal 00 | 10.00 | 7.35 |
| Wheat middlings | 1.13 | 1.45 |
| Animal fat - Poultry | 0.67 | 0.99 |
| Limestone - Coarse | 8.82 | 8.86 |
| Salt | 0.29 | 0.34 |
| Sodium bicarbonate | 0.08 | 0.01 |
| Lysine HCL (79%) | 0.030 | 0.030 |
| Methionine L/DL (99%) | 0.075 | 0.098 |
| Choline-chloride | 0.036 | 0.036 |
| Phytase | 0.003 | 0.003 |
| Premix^1^ | 0.50 | 0.50 |
|  |  |  |
| **Calculated nutrient composition (g/kg)** | | |
| AMEn (kcal/kg) | 2719 | 2715 |
| Moisture | 101 | 111 |
| Ash | 123 | 123 |
| Crude Protein | 160 | 160 |
| Crude Fat | 36 | 36 |
| Crude Fiber | 48 | 44 |
| Starch | 383 | 381 |
| Ca | 36 | 36 |
| P | 4.43 | 4.27 |
| K | 6.87 | 7.10 |
| Na | 1.50 | 1.50 |
| Cl | 2.36 | 2.57 |
| dEB | 175 | 175 |
| US ratio | 5.14 | 4.66 |
| SID LYS | 6.25 | 6.30 |
| SID MET | 3.49 | 3.63 |
| SID CYS | 2.44 | 2.36 |
| SID M+C | 5.94 | 5.99 |
| SID THR | 4.99 | 5.00 |
| SID TRP | 1.52 | 1.54 |
| SID VAL | 6.65 | 6.66 |
| SID ILE | 5.53 | 5.65 |

^1^Contains per kg Vit A: 2500000 IU; Vit D3: 500 000 IU; 25-hydroxycholecalciferol: 3.125 g; Vit E: 6.25 g; Vit K3: 500 mg; Vit B1: 250 mg; Vit B2: 1.25 g; Calcium-D-pantothenate: 1902 mg; Vit B6: 500 mg; Vit B12: 6.25 mg; Niacine: 3.75 g; folic acid: 250 mg; Biotine: 25 mg; Choline chloride: 86.2 g; Iron: 20 g; Copper: 3.75 g; Manganese: 25 g; Zinc: 17.5 g; Iodine: 500 mg; Selenium: 62.5 mg; citric acid: 50 mg; butylhydroxytoluene: 331 mg; propyl gallate: 28 mg; phytase: 150000 FTU/g; β xylanase: 2500 IU/g.
