# Supplementary material for: Short-term dietary deoxynivalenol exposure negatively affects performance, intestinal and reproductive functions in laying hens
Source: Sci Rep. 2026 Apr 11;16:16920. doi: 10.1038/s41598-026-46100-0 (PMC13230629; doi:10.1038/s41598-026-46100-0)
Supplement: Supplementary file 2 — Supplementary Material 2 [file 41598_2026_46100_MOESM2_ESM.docx]

**Supplementary Table 2.** Primers used for the mRNA quantification of genes of interest (GOI) and housekeeping genes (HKG) in the duodenum and eggshell gland of the laying hens.

| **Genes** | **NCBI accession number** | **Primer sequence** | **Annealing T°** | **Reference** |
| --- | --- | --- | --- | --- |
| *HKG* |  |  |  |  |
| GAPDH | NM_204305 | F: GTGTGCCAACCCCCAATGTCTCT  R: GCAGCAGCCTTCACTACCCTCT | 57 | [S1] |
| HPRT | NM_204848.1 | F: CGTTGCTGTCTCTACTTAAGCAG  R: GATATCCCACACTTCGAGGAG | 65 | [S2] |
|  |  |  |  |  |
| *GOI* |  |  |  |  |
| CaBP28K | NM_205513.2 | F: TGTTATGGAGTGCAGGATGG  R: TAGAGCGAACAAGCAGGTGA | 57 | [S3] |
| PMCA1b | XM_046906440.1 | F: TTCAGGTACTCATGTGATGGAAGG  R: CAGCCCCAAGCAAGGTAAAG | 57 | [S3] |
| CaSR | XM_416491.6 | F: GCCAATCTGCTGGGACTCTT  R: CTGATGCTCGTCATTGGGGA | 57 | [S4] |
| NPt2b | NM_204474 | F: ACTGGCTTGCTGTGTTTGC  R: AGGGGCATCTTCACCACTTT | 57 | [S5] |
| XPR1 | XM_422258.6 | F: AACCTGGAGACAACACGAGG  R: CGTTGGTCACCACTTCCTCT | 57 | [S4] |
| OPN | NM_204535.4 | F: AAGAGGCCGTGGATGATGATG  R: ATCCTCAATGAGCTTCCTGGC | 57 | [S6] |
| OCX32 | NM_204534.4 | F: CCAAGAAGAGGACCACAGATT  R: CAACAGCATTGTCCTTCCTTATC | 57 | [S7] |
| OCX36 | XM_025142254.1 | F: CAAGCTGATCTCTGGCTTACTG  R: GGAAGGTGTATGGCTGGATATG | 57 | [S7] |
| OC17 | KF835610.1 | F: CAATGCCTTCGTCTGCAAAG  R: GTGGGTCCGTTTATTGCAGTG | 57 | [S7] |
| VDR | AF011356.1 | F: CGTGGACATTGGGATGATG  R: AGTTTGGGCTTCAGGCTCTC | 57 | [S8] |
| MUC2 | BX930545 | F: ATGCGATGTTAACACAGGACTC  R: GTGGAGCACAGCAGACTTTG | 58 | [S9] |
| CLDN1 | AY750897 | F: GACTCGCTGCTTAAGCTGGA  R: AAATCTGGTGTTAACGGGTG | 58 | [S10] |
| CLDN2 | NM_001277622.1 | F: CAACTGGAAGATCAGCTCCT  R: TGTAGATGTCGCACTGAGTG | 57 | [S4] |
| CLDN3 | NM_204202 | F: AGCCCTCCATCTCAGCAG  R: TTCTCCGCCAGACTCTCC | 57 | [S11] |
| CLDN5 | NM_204201 | F: GTCCCGCTCTGCTGGTTC  R: GCCCTATCTCCCGCTTCTGG | 58 | [S11] |
| GHR | AB075215 | F: TTACTTCAACACATCCTACACC  R: TCATAATCTCTTCCCATCTTCA | 50 | [S12] |
| IGF1R | BQ037565 | F: GTACTTCAGTGCTTCGGATGTG  R: CTTCTTCAGAGTTGGAGGTGCT | 55 | [S12] |
| CASP9 | XM_424580.6 | F: GGAACATTACGCCCGTTCTG  R: ACGATGTCTGACACCCGAAGT | 57 | [S13] |
| BCL2 | NM_205339.2 | F: TCGTCGCCTTCTTCGAGTTC  R: CATCCCATCCTCCGTTGTCC | 57 | [S13] |
| ACC | NM 205505.1 | F: TGTTGAAGGTGACCCGACAG  R: AAGATAGGAGCAGCCCTCCA | 60 | [S14] |
| FABP1 | NM 204192.3 | F: CAGGAGAGAAGGCCAAGTGTA  R: TGGTGTCTCCGTTGAGTTCG | 60 | [S14] |

GAPDH: glyceraldehyde 3-phosphate dehydrogenase; HPRT: hypoxanthine-guanine phosphoribosyl transferase; CaBP28K: Calbidin 28k; PMCA1b: Plasma membrane Ca ATPase; CaSR: Ca sensing receptor; NPt2b: P transporter type II-B Na/P co-transporter; XPR1: xenotropic and polytropic retrovirus receptor 1; OPN: osteopontin; OCX32: ovocalyxin-32; OCX36: ovocalyxin-36; OC17: osteocledin; VDR: vitamin D receptor (VDR); MUC2: mucus production 2; CLDN1: claudin-1; CLDN2: claudin-2; CLDN3, claudin-3; CLDN5: claudin-5; GHR: growth hormone receptor; IGF1R: insulin-like growth factor-1 receptor; Casp9: caspase-9; Bcl2: B-cell lymphoma 2; FABP1: Fatty acid binding protein 1; ACC: acetyl-CoA carboxylase.

**References**

S1. Milanova, A., Santos, R.R., Lashev, L., Koinarski, V. & Fink-Gremmels, J. Influence of experimentally induced *Eimeria tenella* infection on gene expression of some host response factors in chickens. *Bulg. J. Vet. Med*. **19**, 47–56. <https://doi.org/10.15547/bjvm.896>. (2016).

S2. Santos, R.R. et al. Effects of a feed additive blend on broilers challenged with heat stress. *Avian Pathol.* **48**, 582–601. <https://doi.org/10.1080/03079457.2019.1648750>. (2019).

S3. Liu, Y. et al. Effects of feeding strategies on eggshell quality of laying hens during late laying period. *Poult. Sci*. **102**, 102406. <https://doi.org/10.1016/j.psj.2022.102406>. (2023).

S4. Hu, Y.X., van Baal, J., Hendriks, W.H., Duijster, M., van Krimpen, M.M. & Bikker, P. Mucosal expression of Ca and P transporters and claudins in the small intestine of broilers is altered by dietary Ca:P in a limestone particle size dependent manner. *PLoS ONE* **17**, e0273852. <https://doi.org/10.1371/journal.pone.0273852>. (2022).

S5. Wang, X., Li, P., Zhao, J., Jiao, H. & Lin, H. The temporal gene expression profiles of calcium and phosphorus transporters in Hy-Line Brown layers. *Poult. Sci.* **101**, 101736. <https://doi.org/10.1016/j.psj.2022.101736>. (2022).

S6. Shet, D., Ghosh, J., Ajith, S., Awachat, V.B. & Elangovan, A.V. Efficacy of dietary phytase supplementation on laying performance and expression of osteopontin and calbindin genes in eggshell gland. *Anim. Nutr*. **4**, 52-58. <https://doi.org/10.1016/j.aninu.2017.10.004>. (2018).

S7. Zhu, M., Li, H., Miao, L., Li, L., Dong, X. & Zou, X. Dietary cadmium chloride impairs shell biomineralization by disrupting the metabolism of the eggshell gland in laying hens. *J. Anim. Sci*. **98**, skaa025. <https://doi.org/10.1093/jas/skaa025>. (2020).

S8. San, J. et al. Changes in duodenal and nephritic Ca and P absorption in hens during different egg-laying periods. *Heliyon*. **7**, e06081. <https://doi.org/10.1016/j.heliyon.2021.e06081>. (2021).

S9. Forder, R.E.A., Nattrass, G.S., Geier, M.S., Hughes, R.J. & Hynd, P.I. Quantitative analyses of genes associated with mucin synthesis of broiler chickens with induced necrotic enteritis. *Poult. Sci*. **91**, 1335-1341. <https://doi.org/10.3382/ps.2011-02062>. (2012).

S10. Park, C.J. et al. Expression of claudin-1 and -11 in immature and mature pheasant (*Phasianus colchicus*) testes. *Theriogenology* **75**, 445–458. <https://doi.org/10.1016/j.theriogenology.2010.09.012>. (2011).

S11. Ozden, O., Black, B.L., Ashwell, C.M., Tipsmark, C.K., Borski, R.J. & Grubb, B.J. Developmental profile of claudin-3, -5 and -16 proteins in the epithelium of chick intestine. *Anat. Rec.* **293**, 1175–83. <https://doi.org/10.1002/ar.21163>. (2010).

S12. Ni, Y., Zhu, Q., Zhou, Z., Grossmann, R., Chen, J. & Zhao, R. Effect of dietary daidzein on egg production, shell quality, and gene expression of ER-alpha, GH-R, and IGF-IR in shell glands of laying hens. *J. Agric. Food Chem.* **55**, 6997-7001. <https://doi.org/10.1021/jf071085r>. (2007).

S13. Reno K.E., Cloft, S.E. & Wong, E.A. Expression of genes associated with apoptosis in the residual yolk sac during the peri-hatch period of broiler chicks. *Poult. Sci*. **101**, 101966. <https://doi.org/10.1016/j.psj.2022.101966>. (2022).

S.14 Tian, D.L. et al. Effects of lysine deficiency or excess on growth and the expression of lipid metabolism genes in slow-growing broilers. *Poult. Sci.* **98**, 2927-2932. <https://doi.org/10.3382/ps/pez041>. (2019).
